# Autoimmune Hemolytic Anemia (AIHA) Secondary to *Cytomegalovirus* (CMV) Infection in a 2-Month-Old Infant: A Case Report

**DOI:** 10.3390/children10121895

**Published:** 2023-12-07

**Authors:** Stefano Romano, Giuseppe Pepe, Ilaria Fotzi, Tommaso Casini, Elena Chiocca, Sandra Trapani

**Affiliations:** 1 Department of Health Science, University of Florence, 50134 Florence, Italy; giuseppe.pepe@unifi.it; 2 Centre of Excellence, Division of Pediatric Oncology/Hematology, Meyer Children’s Hospital IRCCS, 50134 Florence, Italy; ilaria.fotzi@meyer.it (I.F.); tommaso.casini@meyer.it (T.C.); elena.chiocca@meyer.it (E.C.); 3 Pediatric Unit, Meyer Children’s Hospital IRCCS, 50134 Florence, Italy

**Keywords:** autoimmune hemolytic anemia, cytomegalovirus, children

## Abstract

Autoimmune hemolytic anemia (AIHA) is a rare hematologic disorder in the pediatric population and most cases are associated with microbiological infection. The pathological process is not completely clear, but some evidence suggests immunological dysregulation triggered by bacterial or viral infections. Based on the thermal range of the pathogenic antibody, AIHA can be divided into warm (WAIHA) and cold (CAIHA) groups. *Cytomegalovirus* (CMV) is one of the most common viruses reported as a trigger of AIHA. We present an unusual case of AIHA in a 2-month-old infant positive for both the direct antiglobulin test (C3 complement fraction) and CMV–*Polymerase chain reaction* in blood samples. In this case, the dating of the infection was uncertain, making it impossible to discriminate between congenital flare-up or a primary acute episode, emphasizing the importance of CMV prenatal testing as a screening measure. We adopted multiple therapeutic strategies including steroids (methylprednisolone and prednisone), Intravenous Immunoglobulin, antivirals (ganciclovir and valganciclovir), and red blood cell transfusion.

## 1. Introduction

Autoimmune hemolytic anemia (AIHA) is a rare hematologic disorder, mostly described in adulthood. It is characterized by circulating autoantibodies that bind to the self-antigens of the erythrocyte surface membrane and cause premature red cell destruction [1,2]. The annual incidence of this condition in infants and young children is 0.2 per 100,000/year [3]. The most frequent clinical manifestations include pallor, jaundice, tiredness, or dark urine [4]. The main diagnostic criterion is a positive direct Coombs test (direct antiglobulin test or DAT) result [4], which demonstrates the presence of autoantibodies bound to the erythrocyte surface membrane. Furthermore, the association between AIHA and immune thrombocytopenia, which occurs simultaneously or in succession without any underlying cause, is known as Evans’ syndrome [5].

Based on pathophysiologic mechanisms, AIHA can be divided into warm (WAIHA) or cold (CAIHA), depending on the optimal autoantibody reactivity temperatures [1], respectively, 37 °C for the warm subtype and 4 °C for the cold subtype. WAIHA, occuring more frequently in the infant population, is caused mainly by IgG autoantibodies [1,5], whereas CAIHA is caused by cold agglutinins (IgM autoantibodies that bind at cold temperatures) and complement fractions (especially C3b and C3d) [6]. For the diagnosis of CAIHA, a DAT strongly positive for complement fragments and a cold agglutinin titer of 64 or higher at 4 °C are necessary [6]. Furthermore, there is a mixed subtype in which both IgG and IgM autoantibodies are present.

While AIHA in adults is frequently associated with systemic disorders such as immunodeficiencies, autoimmune diseases, and malignancy, in children it is often secondary to viral and bacterial infections [6,7]. *Cytomegalovirus* (CMV) is a common virus that leads to different clinical manifestations [1], including hematologic disorders. Indeed, CMV infection can cause transient neutropenia and thrombocytopenia or, more rarely, it can lead to AIHA [1]; however, the pathogenetic mechanisms that trigger this uncommon autoimmune response to erythrocyte self-antigens remain unclear [8].

In this report, we describe a case of a 2-month-old infant with AIHA secondary to CMV infection.

## 2. Case

A previously healthy 2-month-old female was admitted to our hospital for new-onset skin pallor, tiredness, and tea-colored urine. Her family denied any other symptoms or the administration of drugs in the days before and she had a negative history of familial hematologic and rheumatological diseases.

At the physical examination, the infant had marked mucocutaneous pallor, low-grade fever, mild tachycardia (heart rate 130 bpm), normal respiratory rate (40 breaths per minute), and oxygen saturation (100%); overall, her general condition was quite good. The cardiac auscultation revealed a systolic murmur of new onset (3/6 Levine), and the liver and spleen were palpable at the costal arch. There was no evidence of jaundice.

Blood tests showed severe anemia (hemoglobin 5.1 g/dL, red blood cell 1.66 × 10^6^, mean corpuscular volume 93.1 fl) and increased reticulocyte percentage (16.9%) with a high absolute number (278.8 × 10^9^/L). The counts of white blood cells (11.3 × 10^9^/L, neutrophils 29.7%, lymphocytes 53.5%, monocytes 6.2%), and platelets (675 × 10^9^/L) were normal. Biochemical investigations showed increased total bilirubin (2.0 mg/dL), mostly the indirect fraction (1.4 mg/dL), and a mild increase of lactate dehydrogenase (LDH) (405 UI/L) with normal creatinine and transaminases.

In suspicion of AIHA, the Coombs Test was performed, revealing a positive DAT with the presence only of complement fractions (C3b and C3d) on erythrocyte surface membranes without anti-IgG, while the indirect antiglobulin test (IAT) was negative. These data, together with clinical and laboratory signs of hemolysis, led us to confirm the diagnosis of AIHA but did not allow us to classify it in either the warm or cold subtype because IgG autoantibodies and cold agglutinins were both negative.

On day zero, according to the hematologist specialist, we started preheated erythrocyte transfusion support and steroid therapy with oral prednisone (2 mg/kg/day).

Despite CMV-*PCR* on Dried Blood Spot (DBS) test being negative, infectious investigations revealed CMV *PCR* positivity on the nasopharyngeal swab (9140 viral load/mL), blood (1300 viral load/mL), and urine (57,000 viral load/mL). Specific serological tests showed negative IgM and positive IgG with high avidity; these data did not allow us to precisely date the infection. Ophthalmological, audiometry, and encephalic trans fontanel ultrasound, performed in suspicion of congenital infection, were all negative. In order to exclude other infectious diseases, *PCR* for Epstein-Barr virus, adenovirus, enterovirus, and parechovirus on nasopharyngeal swab were performed and resulted negative. Hemoglobin level increased to 6.7 g/dL on day one (the day after the first red cell transfusion), then decreased again to 5.9 g/dL. On day two, antiviral therapy with intravenous (IV) ganciclovir was initiated (6 mg/kg/dose twice a day).

On day three, due to poor compliance with oral therapy, steroid treatment was shifted to IV methylprednisolone (MPD) (2 mg/kg/day). On day five, antiviral treatment was shifted to oral valganciclovir (16 mg/kg/dose twice a day). As hemoglobin levels did not increase, on day six a second pre-heated red cell transfusion was performed. On day seven a dose of 1 g/kg of IV immunoglobulin (IVIG) was given. IV steroid therapy was continued until day 10, when treatment was shifted to oral prednisone administration at the same dosage.

Hemoglobin started to progressively increase until 9.3 mg/dL on day 11, with a consistent decrease in indirect bilirubin and LDH from day three, suggesting that the hemolytic process was stopped. During hospitalization, the patient was always able to breastfeed without the need for enteral tube nutrition. Pre-discharge laboratory tests showed total bilirubin 0.5 mg/dL, indirect bilirubin 0.3 mg/dL, LDH 335 UI/L, and reduced levels of reticulocyte count (4.9%). Due to the improved clinical conditions and increased hemoglobin levels, on day 11 the patient was discharged with the recommendation of continuing both steroid therapy with prednisone and antiviral therapy with valganciclovir. After discharge, the patient’s health assessments were strictly followed up in the Hematology ward of our hospital. At the first and second clinical and laboratory checks (on days 15 and 23, respectively), hemoglobin levels showed an increasing trend (9.4 g/dL and 11.2 g/dL). At the third check (on day 33), because of stable levels of hemoglobin (10.9 mg/dL), a slow tapering of steroid therapy was started, initially at the dose of 1.7 mg/kg/day, before being reduced to 1.3 mg/kg/day. Working according to the decision of infectious disease specialists, it was decided to continue antiviral therapy with valganciclovir alongside steroid therapy. During tapering down, hemoglobin levels showed a fluctuating trend, associated with a mild increase in hemolytic factors (bilirubin levels and reticulocyte count). At the time of the last control (after 4 months), when steroid therapy was tapered down to 1 mg/kg/day, hemoglobin was 10.1 g/dL, total bilirubin was 2.5 mg/dL, LDH 507 UI/L, and there was a reticulocyte count of 4%.

## 3. Discussion

AIHA in children, unlike in adults, is most often an isolated phenomenon and not related to other autoimmune, malignant, or inflammatory conditions or drugs, although all these associations are possible.

About 40 years ago, Heisel was one of the first authors to propose a subdivision into two varieties of AIHA in children based on prognostic factors: on one hand, patients aged 2–12 years old, with a sudden onset of symptoms, low reticulocyte counts, and normal platelet counts, tending to successfully respond to steroids [9]; on the other hand, children less than 2 or older than 12 years old, with a long-lasting onset of symptoms, high reticulocyte counts, and decreased platelet counts, tending to become chronic and have a variable response to steroids and usually requiring other treatments [9]. Nowadays, AIHAs are classified based on the thermal range of the pathogenic antibody (warm antibody AIHA and cold antibody AIHA) and whether they are associated with another disease or not (primary or secondary). Warm AIHA is most often precipitated by non-specific viral infections, whereas CAIHA is commonly associated with *Mycoplasma pneumoniae* infection and, more rarely, with mononucleosis and chickenpox [10].

In our case, AIHA was related to CMV infection, diagnosed via the detection of CMV *PCR* on blood, urine, and nasopharyngeal swabs. However, it was not possible to classify it into the warm or cold subtypes because DAT showed the presence only of complement fractions on the erythrocyte surface, with negative autoantibodies IgG and cold agglutinins IgM. We suppose that CMV infection most likely triggered an aspecific immune response with the activation of the complement system that nonspecifically adhered to the erythrocyte surface membrane, determining the hemolytic process. CMV has been implicated in the pathogenesis of AIHA in children, although the exact pathogenetic mechanism remains unknown [1,8]. It is assumed to be the trigger of an autoimmune dysregulated response to erythrocyte self-antigens. One possible mechanism, common to other autoimmune conditions, is molecular mimicry [2]: viral epitopes, which share structural similarities with self-antigens, lead to antibody cross-reactivity. The dysregulated response could also be explained by the loss of tolerance of autoreactive B- or T-cells, activated by cytokines and chemokines released by antigen-presenting cells (APCs), that are attracted to the site of CMV infection [11]; this phenomenon is called “bystander activation” [11].

In our patient, it was not possible to exactly date the time of the infection because CMV-*PCR* on the DBS test was negative, which excluded a congenital infection. However, the sensitivity of this test was low. We believe a post-natal infection was more likely; furthermore, the infant was fed with maternal breast milk, which is known to be a primary cause of postnatal CMV infection [12].

In the largest pediatric cohort with AIHA (265 children), described by Aladjidi et al. [5], 24% of them had CAIHA, while the others belonged to the warm or mixed subtype. Furthermore, the hemolytic process was associated with CMV infection in only six patients (2.3%). In this study, the authors identified the IgG/IgG + C3d DAT as an unfavorable prognostic factor: in fact, the rate of continuous complete remission was 18% in the warm/mixed subtype and 71% in the cold one [5]. In the study of Naithani et al. [13], conducted on 26 children with WAIHA, only one patient (3.8%) had CMV as the causative agent. Thatikonda et al. [14] described 50 pediatric patients with AIHA, of whom 35% had CAIHA, and only one (2%) had hemolytic anemia secondary to CMV infection. In another study by Abdel-Salam et al. [4] on 50 patients, 9 (18%) had a recent CMV infection. Overall, these studies suggest that AIHA secondary to CMV infection is a rare occurrence however, CMV serology is not regularly obtained in patients with hemolysis, and so the incidence of this condition can be underestimated [8].

CMV can often cause warm AIHA (IgG+) and, more rarely, cold (C3+) or mixed subtypes (IgG+, C3+). The C3 isolated CAIHA is usually associated with a better prognosis, a short follow-up period, and a two-year complete remission rate of up to 71% [5]. The mixed subtype, instead, tends to evolve chronically, with the risk of periodic hemolytic crises [1]. In the literature, only a few case reports of AIHA associated with CMV infection in infants less than 1 year old have been published (Table 1). Khalifeh et al. [1] described a 6-month-old infant with severe warm AIHA who responded well to steroids and IVIG, without red cell transfusions. Vijayaran et al. [15] reported two cases of infants, a 6-month-old and a 5-month-old, with severe anemia and a CMV infection, respectively, both treated with antiviral therapy. The first had chronic and refractory mixed AIHA. This was initially treated with IVIG, steroids, and ganciclovir, and then, due to relapses upon stopping steroid treatment, required a second-line treatment with rituximab, and later mycophenolate mofetil (MPM). The second infant, instead, showed a negative Coombs test result and was successfully treated with only ganciclovir. Murray et al. [2] described two other cases: an 11-month-old infant with WAIHA and a 4-month-old baby with mixed AIHA; both were successfully treated with steroids and IV CMV immune globulins. Yamamoto et al. [16] reported the case of a 31-day-old infant with WAIHA exacerbated by CMV infection, whose mother had systemic lupus erythematosus and hemolytic anemia, that was treated with blood transfusions and antiviral therapy. In children, AIHA secondary to CMV infection is mostly represented by the warm or mixed subtype, as reported in the above-mentioned case reports [1,2,15]; however, in large cohorts of children with AIHA [4,5,14], there is no explanation of which subtype patients with CMV-associated AIHA belong to.

In patients with AIHA, the first-line treatment consists of steroids: for severe cases with rapidly evolving hemolysis, pulse high-dose methylprednisolone (30 mg/kg/day for 3 days) is recommended [17]. Children with mild or moderate anemia can be treated with oral prednisone (2 mg/kg/day), followed by a slow taper [1]. IVIG (1 g/kg/day) for 2 days is usually recommended in children with a partial response to steroids [4]. Second-line treatments, such as azathioprine, cyclosporine A, mycophenolate mofetil (MPM), and rituximab, are indicated in patients with resistant AIHA or relapse after stopping steroids [4]. In our patient, as soon as AIHA was diagnosed, first-line therapy with prednisone was initiated; working according to the recommendations of the hematologist specialist, it was first decided to start with oral steroids instead of IV upfront because of stable vital signs and good general conditions. Afterward, due to poor compliance with oral therapy, the treatment was shifted to IV methylprednisolone. With hindsight, it would have been more appropriate to start with IV therapy upfront. Due to the partial response, we administered one single infusion of IVIG. At the same time, due to the positivity of *PCR* CMV, we considered the infection to be the main contributing cause in the pathogenesis of AIHA; therefore, working according to the recommendations of infectious disease specialists, we started antiviral therapy, first with IV ganciclovir, and then with oral valganciclovir. We decided to continue this therapy alongside steroids to avoid reactivation of the CMV infection. In previous case reports of infants with AIHA related to CMV treated with ganciclovir/valganciclovir, it is not specified how long the antiviral therapy was administered [15,16].

There are no standardized prognostic factors able to predict the response to first-line treatments and the evolution of AIHA in children. Aladjidi et al. [5], in the largest cohort of pediatric patients with AIHA, indicated the type of AIHA as being the main prognostic factor; in fact, the warm and mixed subtypes were significantly associated with the lack of complete remission achievement after 1 month of steroid treatment and with a lower rate of continuous complete remission compared to the better outcomes obtained using CAHIA [5]. In contrast, Heisel et al. had previously described four major prognostic factors, independently of the subtype: age, platelet counts, reticulocyte counts, and onset of symptoms [9].

Our patient, according to Heisel et al. [9], had two favorable prognostic factors (normal platelet counts and sudden onset of symptoms) and two unfavorable factors (age < 2 years old and high reticulocyte counts). Initially, she required two red cell transfusions and did not completely respond to steroid therapy (oral and IV), needing one infusion of IVIG; at discharge, her hemoglobin was stable without signs of hemolysis. Thereafter, hemoglobin levels started progressively to increase, and steroid therapy was slightly tapered down. So far (about 4 months from the beginning of steroid therapy), she has had a fluctuating course, with quite stable hemoglobin levels but fluctuating levels of hemolytic factors.

The main limitations of our study are the inability to classify the AIHA subtype and the short follow-up period. In fact, the DAT showed positivity only for complement fractions but did not allow us to classify the AIHA in either the warm or cold subtype. The follow-up period was about 4 months, and the patient is still undergoing steroid therapy, which is being tapered down over the course of 6 months; at the end of this period, we will know if she belongs to the low-risk group or if she will need second-line therapeutic strategies.

This case demonstrates that many elements contribute to the clinical course of AIHA in children and that there are not yet shared prognostic factors.

## 4. Conclusions

We described an unusual case of AIHA secondary to CMV infection in a 2-month-old girl treated with steroids, IVIG, ganciclovir/valganciclovir, and who needed two red blood cell transfusions. This case emphasizes the wide spectrum of AIHA presentation and how it can sometimes need a combination of therapeutic strategies.

## Figures and Tables

**Table 1:** Case reports of AIHA associated to CMV infection in children under 1 year.

Reference	Age	Type of AIHA	Treatment
Khalifeh et al. [1]	6 months	Warm AIHA	Steroids and IVIG
Vijayaran et al. [15]	6 months	Mixed AIHA	IVIG, steroids, ganciclovir, rituximab and MPM
	5 months	Negative Coombs test	Ganciclovir
Murray et al. [2]	11 months	Warm AIHA	Steroids and IV CMV immune globulin
	4 months	Mixed AIHA	
Yamamoto et al. [16]	31 days	Warm AIHA	Antiviral therapy
Current case	2 months	AIHA	Steroids, IVIG and antiviral therapy

## Data Availability

The data presented in this study are available in article.
